# The relationship between orthostatic hypotension and cognitive impairment in Parkinson’s disease: a systematic review and meta-analysis

**DOI:** 10.3389/fmed.2025.1659043

**Published:** 2025-09-09

**Authors:** Yiping Liu, Yifan Jiang, Jingyi Wang, Yan Shi

**Affiliations:** School of Medicine, Tongji University, Shanghai, China

**Keywords:** Parkinson’s disease, orthostatic hypotension, cognitive impairment, cognitive domains, meta-analysis

## Abstract

**Objective:**

Orthostatic hypotension (OH) and cognitive impairment are prevalent non-motor symptoms in Parkinson’s disease (PD). Although numerous epidemiological studies have explored the association between OH and cognitive impairment, the findings remain controversial. This study aims to systematically evaluate the relationship between OH and cognitive function in patients with PD and to investigate the impact of OH on different cognitive domains.

**Methods:**

Databases, including the Chinese National Knowledge Infrastructure (CNKI), Wanfang Database, SinoMed, VIP (Database of Chinese Scientific and Technical Periodicals), PubMed, Embase, Cochrane Library, Web of Science, ProQuest, Scopus, and Ovid, were searched for eligible publications from their inception to July 2025. After literature screening and quality evaluation based on inclusion and exclusion criteria, meta-analysis, heterogeneity testing, sensitivity analysis, and subgroup analysis were conducted using Review Manager 5.4 software. Publication bias analysis was assessed using Stata software.

**Results:**

Thirteen studies with a total of 1,417 participants were ultimately included, comprising 552 Parkinson’s patients with OH (PD-OH group) and 865 Parkinson’s patients without OH (PD-NOH group). Compared to the PD-NOH group, the global cognitive score of the PD-OH group was significantly lower [SMD = −0.62, 95%*CI* [−0.78, −0.46], *p* < 0.01]. In terms of cognitive domains, the PD-OH group showed the following results: memory: SMD = −0.12, 95% *CI* (−0.64, 0.17), *p* = 0.25; executive function: SMD = −0.29, 95% *CI* (−0.50, −0.07), *p* < 0.01; verbal ability: SMD = −0.35, 95% *CI* (−0.65, −0.04), *p* < 0.01; attention: SMD = −0.12, 95% *CI* (−0.33, 0.09), *p* = 0.27; and visuospatial function; SMD = −0.40, 95% *CI* (−0.61, −0.18), *p* < 0.01. PD patients with OH did not exhibit significant cognitive impairment in the attention and memory domains but showed marked cognitive deficits in the executive function, verbal ability, and visuospatial function.

**Conclusion:**

This meta-analysis indicates that cognitive function decline in PD patients is associated with OH. Patients with OH have lower global cognitive scores compared to those without OH, particularly demonstrating significant deficits in executive, verbal, and visuospatial functions, especially in those with a long disease duration. Clinicians should be vigilant about these potential cognitive deficits and consider comprehensive cognitive assessments and targeted interventions for PD patients experiencing OH.

## 1 Introduction

Parkinson’s disease is a complex, age-related neurodegenerative disorder characterized by dopamine deficiency and a spectrum of motor and non-motor deficits (1, 2). Orthostatic hypotension, defined as a sustained reduction of systolic blood pressure ≥20 mmHg or diastolic blood pressure ≥10 mmHg within 3 min of a patient moving from a supine to a standing position, with or without cerebral hypoperfusion symptoms (3), ranks among the most prevalent non-motor manifestations in PD, with an estimated prevalence reaching 52.8% (4, 5). Common symptoms include dizziness, fatigue, neck pain, presyncope, and syncope (6, 7).

Cognitive impairment represents another debilitating non-motor symptom of PD, contributing significantly to disability burden with limited therapeutic options (8, 9). Notably, OH and cognitive impairment frequently co-occur in PD patients, collectively exerting a substantial negative impact on disease progression. The potential association between OH and cognitive decline in PD is hypothesized to stem from several interrelated pathophysiological mechanisms: (1) Cerebral Hypoperfusion: Transient systemic blood pressure drops during postural changes may compromise cerebral blood flow, particularly in vulnerable watershed areas and regions critical for higher cognition (e.g., prefrontal cortex and hippocampus). This recurrent hypoperfusion could induce chronic ischemic damage, neuronal dysfunction, and accelerate neurodegeneration (10) and (2) Shared Neurodegenerative Pathology: OH in PD often reflects underlying autonomic nervous system (ANS) degeneration, driven by the hallmark PD pathology of alpha-synuclein aggregation. This synucleinopathy affects key brainstem autonomic nuclei (e.g., locus coeruleus and dorsal motor nucleus of the vagus) and peripheral ganglia, disrupting cardiovascular regulation. Crucially, the neurodegenerative process extends beyond the ANS to involve cortical and subcortical cognitive structures (11). This finding suggests that OH and cognitive impairment may often represent parallel manifestations of a widespread synucleinopathy rather than a simple cause–effect relationship. Understanding these mechanisms provides essential context for interpreting the relationship between OH and cognitive function in PD.

Despite this pathophysiological rationale, the clinical correlation between OH and cognitive impairment in PD remains controversial (4, 12). While some studies identify OH as a risk factor for cognitive decline (13–17), others report no direct association (18–21). Furthermore, the specific cognitive domains most vulnerable to OH in PD are poorly defined. Given these unresolved questions and the emergence of new research, we aimed to systematically analyze the relationship between OH and global cognitive dysfunction in PD through comprehensive literature retrieval and meta-analysis. In addition, we aimed to explore the impact of OH on distinct cognitive domains. The findings are intended to provide an evidence-based rationale for the early identification of patients at high risk of progressing to PD-mild cognitive impairment (PD-MCI) or PD dementia (PDD).

## 2 Materials and methods

### 2.1 Literature search strategy

The protocol of this systematic review was developed in accordance with the Preferred Reporting Items for Systematic Reviews and Meta-Analyses (PRISMA) guidelines (22) and registered with the INPLASY (INPLASY202490130). A systematic literature search was conducted in the Chinese National Knowledge Infrastructure (CNKI), Wanfang Database, SinoMed, VIP (Database of Chinese Scientific and Technical Periodicals), PubMed, Embase, Cochrane Library, Web of Science, ProQuest, Scopus, and Ovid databases for studies in English or Chinese. The search terms included: “Orthostatic hypotension” OR “Postural Hypotension” OR “Hypotension, Postural” and “Parkinson’s” OR “Parkinson” OR “Parkinsonism” OR “Paralysis Agitans” and “cognitive dysfunctions” OR “cognitive impairment” OR “Mental Deterioration.” Additionally, the reference lists of the retrieved studies were also reviewed to identify any relevant studies not captured through the database search.

### 2.2 Inclusion and exclusion criteria

The inclusion criteria were as follows: (1) Study type: case–control studies published in English or Chinese. (2) Participants: Patients diagnosed with PD according to study-defined diagnostic criteria, such as the UK PD Society Brain Bank criteria (23) or the Movement Disorder Society (MDS) clinical diagnostic criteria for PD (24) or the Chinese diagnostic criteria for Parkinson’s disease (25), regardless of age, gender, ethnicity, or disease duration. (3) PD was combined with OH in the experimental group. (4) Control group: The experimental group comprised patients with PD combined with OH, while the control group comprised patients with PD without OH. OH was defined as a drop of ≥20 mmHg in systolic blood pressure and/or ≥10 mmHg in diastolic blood pressure within 3 minutes of standing. (5) Outcome indicators: Overall cognitive function was determined using the Mini-Mental State Examination (MMSE) (26) or the Montreal Cognitive Assessment (MoCA) (27). Clinical measurements, including mean, standard deviation, and number of participants, were reported. Cognitive domains and their corresponding assessment scales were determined based on the diagnostic criteria for mild cognitive impairment established by the International Parkinson and Movement Disorder Society.

The exclusion criteria were as follows: (1) Studies from which effective outcome data could not be extracted; (2) Studies involving secondary parkinsonism, progressive supranuclear palsy (PSP), multiple system atrophy (28), and other parkinsonism syndromes.

### 2.3 Data extraction

Two review authors independently extracted data. A third author was involved in case of disagreements.

We divided the data into two groups: PD with OH and PD without OH. The main extracted data included the following: (1) the name of the first author, (2) publication year, (3) country in which the study was conducted, (4) study quality, (5) sample size, (6) gender, (7) disease duration, (8) mean age, (9) OH measurement method, (10) education years, (11) PD H–Y stage, (12) UPDRS III, (13) levodopa equivalent dose, (14) global cognitive assessment scale, and (15) cognitive domain test scale.

### 2.4 Literature quality evaluation

The quality of the included studies was assessed using the Newcastle–Ottawa scale (NOS). Case-control studies were evaluated using an 8-item scale, which rated the following domains on a 9-star system: participants, group selection, comparability, and exposure evaluation (29). Each evaluator assessed the risk of bias in the included studies and, in cases of disagreement, consensus was reached through discussion or third-party resolution.

### 2.5 Statistical analysis

This meta-analysis was conducted using RevMan 5.4 software. For continuous outcome measures—including MMSE, MoCA, and other neuropsychological test scores (reported as mean ± standard deviation)—the standardized mean difference (SMD) with 95% confidence intervals was calculated as the effect size metric. First, Cochran’s Q-test (*χ^2^*) was used to assess heterogeneity among included studies, with the magnitude quantified using the *I^2^* statistic. When *I^2^* ≤ 50% and *p* > 0.10, heterogeneity was considered low, and a fixed-effect model was applied to calculate the pooled results. When *I^2^* > 50% or *p* ≤ 0.10, indicating substantial heterogeneity, a random-effects model was used for pooled analysis. To evaluate the stability of the results, we performed sensitivity analyses. We also conducted subgroup analyses to explore sources of high heterogeneity. Finally, Egger’s test and the funnel plot were used to assess publication bias.

## 3 Results

### 3.1 Literature search

**Figure 1:**
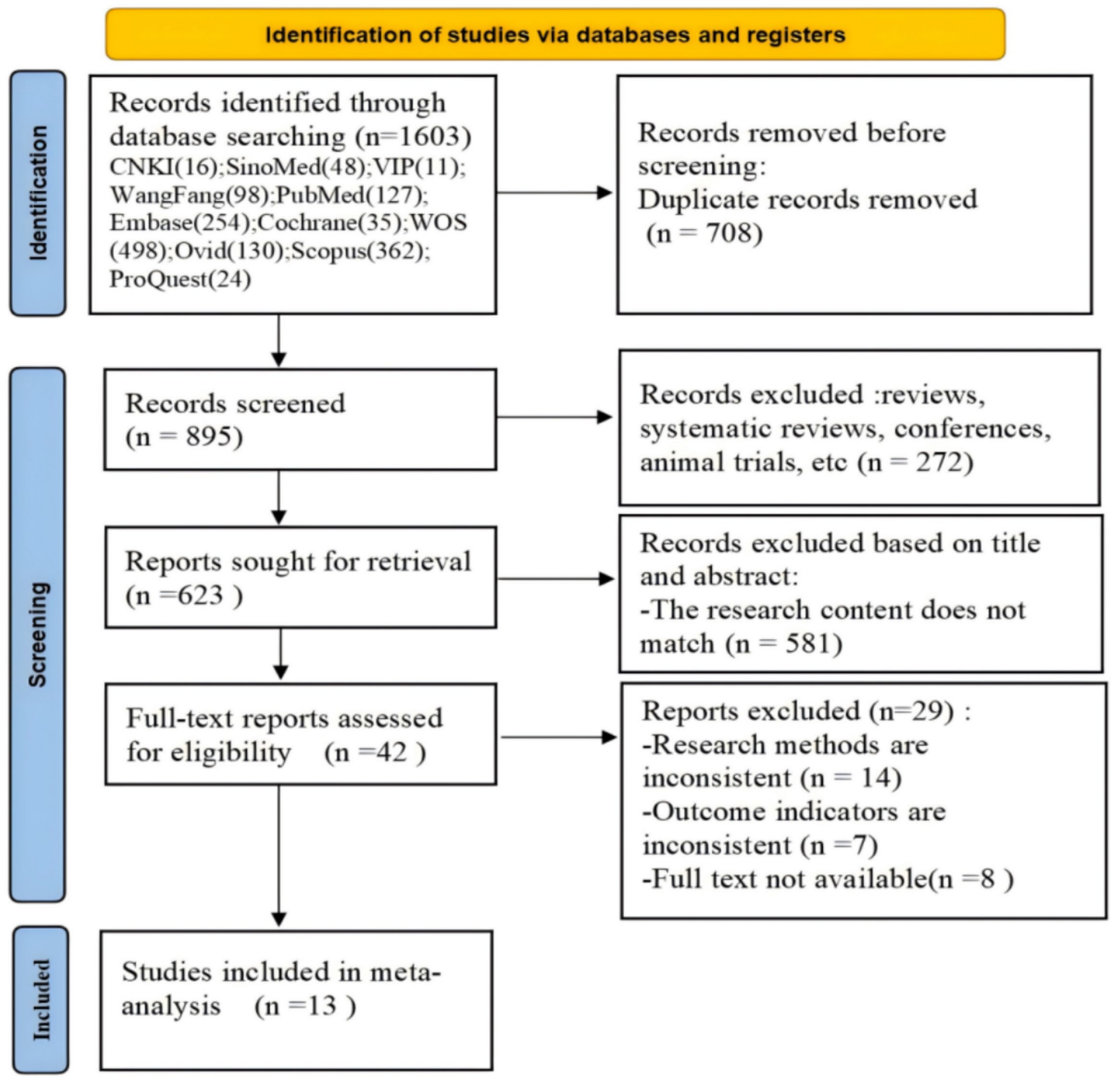
Flow diagram of the literature search.

A total of 1,603 articles were retrieved in this literature search, of which 895 articles were produced after the removal of duplicate articles. After preliminary screening, 623 articles were identified. Of these, 581 articles were excluded because they did not meet the inclusion criteria. Finally, 13 studies of good methodological quality were included. The flowchart of this literature search is shown in Figure 1.

### 3.2 Study characteristics

The main characteristics of selected studies are shown in Table 1. The 13 studies included a total of 1,417 participants (30–42), among whom there were 552 PD patients with OH and 865 PD patients without OH. This meta-analysis included eight Chinese studies and five English studies. The specific cognitive domain tests included in this meta-analysis are shown in Table 2. According to the NOS evaluation scale, the literature scores are above 6 points, which can be included in this systematic evaluation.

**Table 1:** Characteristics of the 13 studies included in this meta-analysis.

Author, Year	Country	OH test method	Sample size (*n*)	Male sex (%)	Mean Age	Disease duration (mean, year)	Education years (mean)	H–Y stage (mean)	Levodopa equivalent dose (mean, mg)	UPDRS III (mean)	Global cognitive assessment scale	Study quality
Allcock, L. M. 2006	UK	AST	175	109 (62%)	70.8	4.25	NA	NA	350	17.75	MMSE	7
Centi, Justin 2017	USA	HUTT	37	22 (60%)	64.95	6.2	17.2	2	552	NA	MMSE	6
Chenfei Liu 2022	China	HUTT	54	23 (43%)	66.67	6.5	9.19	2.5	514.57	34.36	MMSE+MoCA	8
Hohler, Anna D. 2012	USA	AST	44	27 (61.4%)	NA	NA	13.1	4	NA	NA	MMSE	6
Li, L. 2019	China	AST	150	78 (52%)	66.16	4.25	8.94	2	376.45	29	MoCA	8
Lianchang Wang 2022	China	AST	119	74 (62%)	65.85	7.62	11.25	NA	NA	NA	MMSE+MoCA	8
Longardner, Katherine 2020	USA	AST	226	149 (65%)	67.9	5.7	16.3	2.25	NA	28.1	MoCA	7
Meng, Yuanyuan 2024	China	AST	171	102 (60%)	65.25	7	NA	3.5	NA	22.5	MMSE+MoCA	8
Pilleri, M. 2013	Italy	HUTT	48	26 (54%)	65.28	11.62	11.19	2.71	950.5	37.6	MMSE	8
Wanjun Mi 2021	China	AST	169	100 (59.17%)	64.27	5.42	10.16	1.98	NA	NA	MMSE	8
Xue, X. 2023 (1)	China	AST	79	35 (51%)	62.9	4.22	11.12	2.27	422.66	32.67	MMSE	8
Xue, X. 2023 (2)	China	AST	31	14 (45%)	59.65	3.37	10.89	1.99	275.9	28.8	MMSE+MoCA	8
Yin, K. 2022	China	AST	116	67 (58%)	66.5	3.5	8	2.5	343.75	29	MMSE+MoCA	8

USA, United States of America; UK, United Kingdom; AST, active standing test; HUTT, head-up tilt test; H–Y stage, Hoehn–Yahr stage; UPDRS III, Unified Parkinson’s Disease Rating Scale III; NA, not available.

**Table 2:** Cognitive domain tests included in the primary studies.

Study	Executive function and working memory	Attention	Memory	Verbal	Visuospatial function
Allcock, L. M. 2006	Numeric working memory	SRT, CRT, Digit vigilance	Verbal memory	–	Visual memory
Centi, Justin 2017	A Stroop color test	Digit-Span Forward	CERAD total score	Semantic Fluency	Visual Dependence
Pilleri, M. 2013	Reproduction	Digit-Span Forward	Delayed recall	Semantic	ROCF
Xue, X. 2023	–	–	MoCA	–	–
Yin, K. 2022	MMSE	MMSE	MMSE	MMSE	MMSE

### 3.3 Meta-analysis results

#### 3.3.1 Heterogeneity test

**Figure 2:**
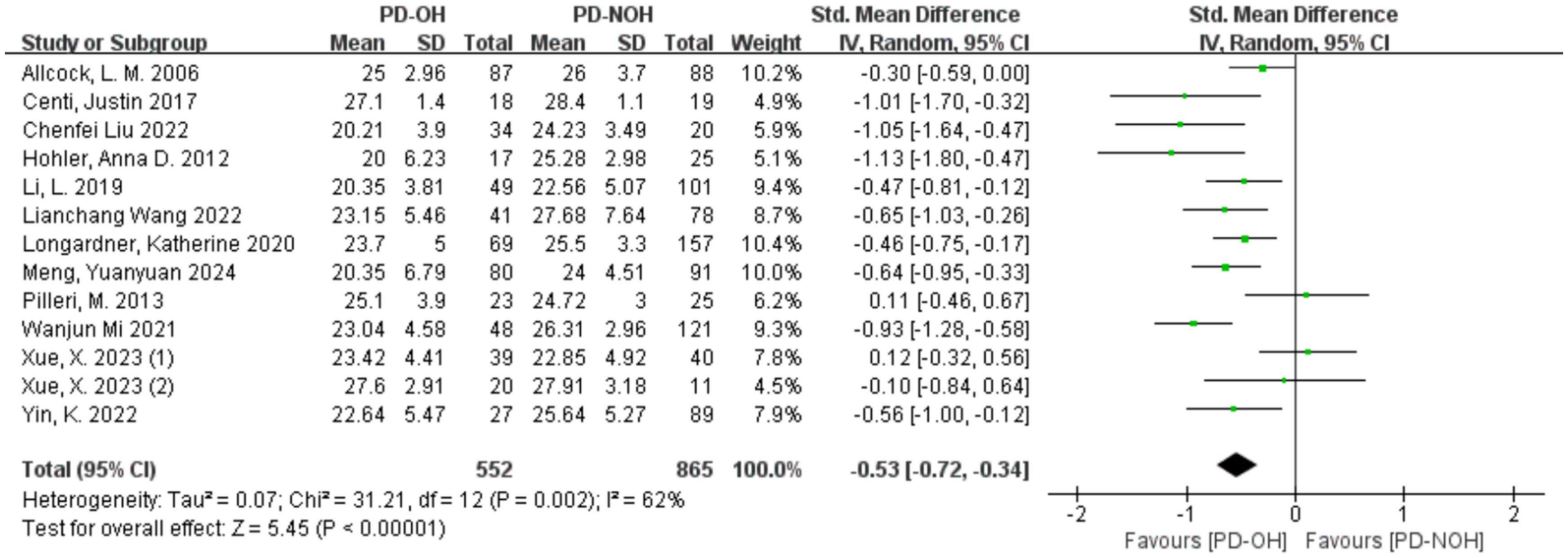
Forest plot for the comparison of global cognitive function in PD patients with corresponding 95% *CI.*

Heterogeneity testing across the 13 included studies revealed substantial heterogeneity (*I^2^* = 62%, *p* = 0.002), exceeding acceptable thresholds (*p* < 0.10). This statistically significant variation necessitated investigation of heterogeneity sources, as shown in Figure 2.

#### 3.3.2 Sensitivity analysis to identify sources of heterogeneity

**Figure 3:**
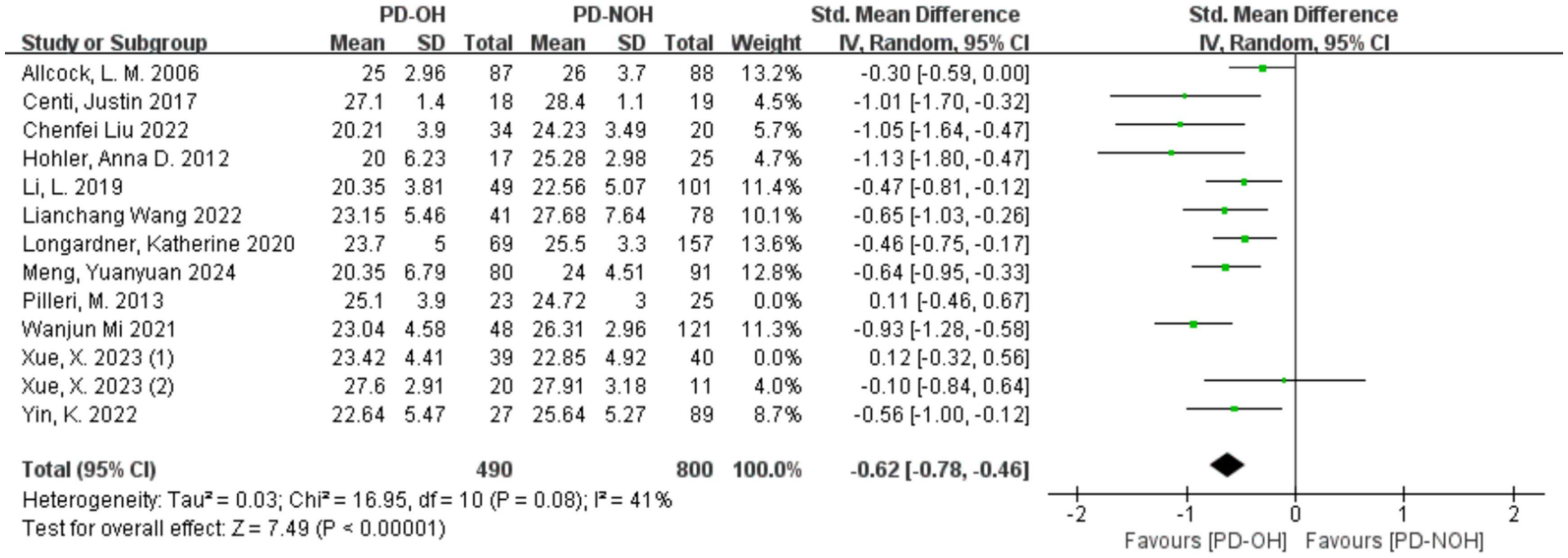
Forest plot for the comparison of global cognitive function in PD patients with corresponding 95% *CI.*

Sensitivity analysis of the 13 included studies showed that Xue (40) (1) and Pilleri et al. (37) had a significant influence on heterogeneity. After removing these two studies, the heterogeneity decreased, with *I*^2^ = 41% < 50%, *p* = 0.08, indicating a lower level of heterogeneity than before. After exclusion, a random effect model was used for the meta-analysis. The SMD value of the 11 studies was −0.62, and the 95% confidence interval was [−0.78, −0.46], which was statistically significant (Z = 7.49, *p* < 0.01), indicating that the global cognitive function score of patients in the PD-OH group was lower than that in the PD-NOH group, as shown in Figure 3. In the study by Pilleri et al. (37), the small sample size and exclusion of PD patients with a disease duration <5 years may have resulted in the inclusion of a PD cohort with a stronger cognitive reserve (mean MMSE >24). Additionally, 39% of OH + patients had co-existing supine hypertension (SH); this chronic hypertensive state may attenuate the acute hypoperfusion effects of OH through vascular structural adaptations, contributing to discrepancies across studies. In the study by Xue et al. (41) (1), a significant intergroup difference in years of education (3.6 years) was observed. Longer education may partially compensate for memory impairment, leading to effect size fluctuations between studies. Furthermore, failure to control for confounding factors such as supine hypertension contributed to the inconsistencies in the results.

#### 3.3.3 Meta-analysis of the relationship between PD orthostatic hypotension and impairment of different cognitive domains

**Figure 4:**
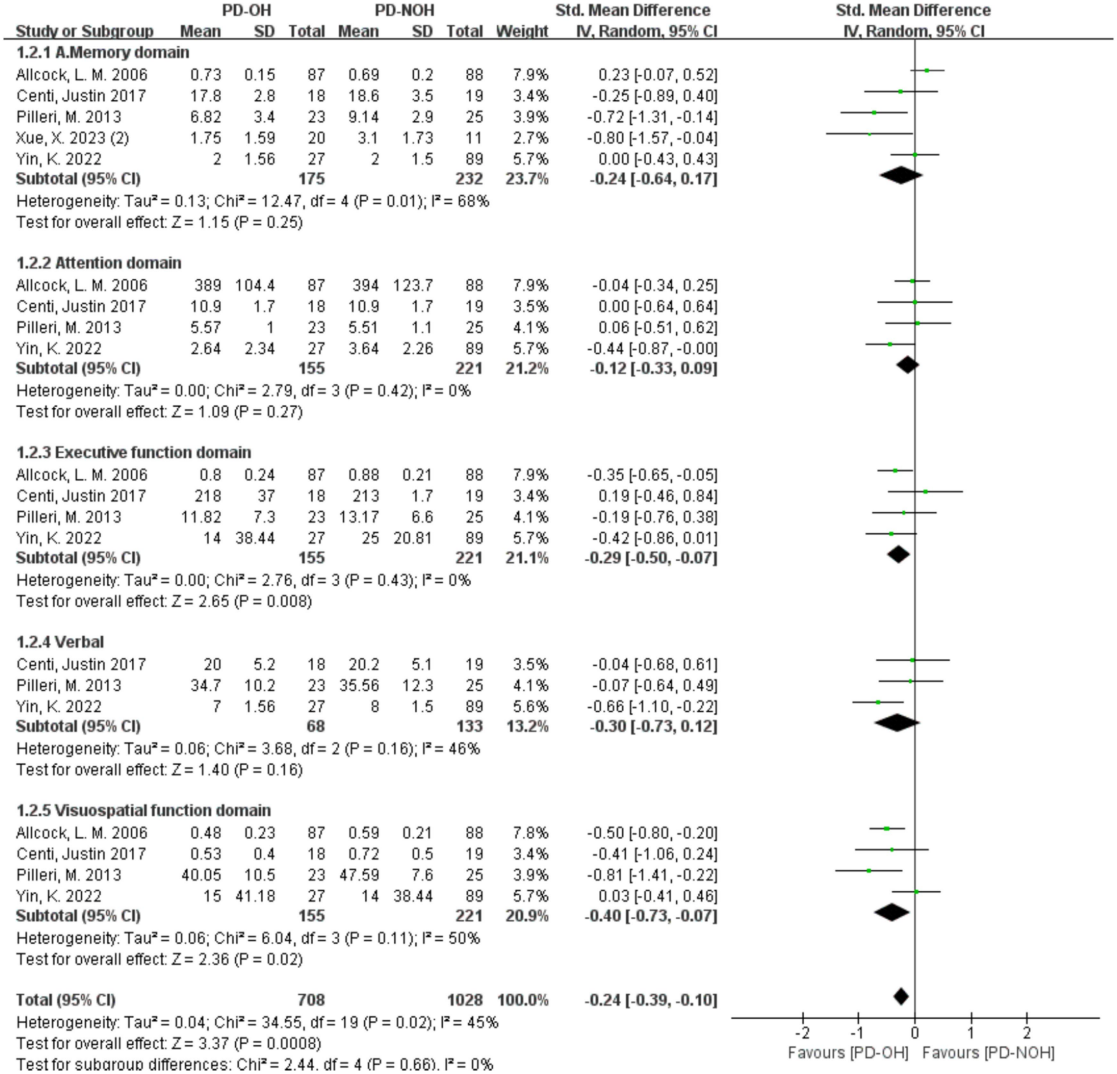
Forest plots of the meta-analysis of the cognitive domain difference between PD patients.

Heterogeneity was acceptable (*I^2^* < 50%) in all domains except memory (*I^2^* = 68%); fixed-effect models were thus applied for non-memory domains, with a random-effects model used for memory (Figure 4). The results showed that the PD-OH group had the following results in each cognitive domain: Memory domain: SMD = −0.12, 95% *CI* (−0.64, 0.17), *p* = 0.25; execution function domain: SMD = −0.29, 95% *CI* (−0.50, −0.07), *p* < 0.01; verbal domain: SMD = −0.35, 95% *CI* (−0.65, −0.04, *p* < 0.01); attention domain: SMD = −0.12, 95% *CI* (−0.33, 0.09), *p* = 0.27; and visuospatial function domain: SMD = −0.40, 95% *CI* (−0.61, −0.18), *p* < 0.01. Significant cognitive impairment was observed in the executive, verbal, and visuospatial domains (*p* < 0.01), but not in attention or memory.

#### 3.3.4 Subgroup analyses

The subgroup analyses stratified by ethnicity, gender, H–Y stage, education years, and disease duration years are shown in Table 3. All subgroup stratifications were performed at the study level, utilizing aggregate characteristics reported for the entire study population. The chi-square test, based on the Q statistic, was used to determine whether, within a given subgroup, there remained a significant difference between PD-OH and PD-NOH, represented by a *P*_heterogeneity_ statistic. A separate *P*_different_ statistic was calculated to determine whether sub-group stratification significantly enhanced statistical differences between PD-OH and PD-NOH groups across studies. The subgroup analysis indicated that, based on ethnicities, significant differences existed between the PD-OH group and the PD-NOH group both in Asian populations and in European and American populations, with the PD-OH group consistently showing lower mean scores than the PD-NOH group. Although moderate heterogeneity was present within both subgroups, no statistical significance was observed in the differences between subgroups, suggesting that geographical factors may not be the crucial factor influencing the disparities between PD-OH and PD-NOH, or the impact of geographical variations on the results is not pronounced in this study. In subgroups stratified by H–Y stage, statistically significant differences in cognitive function between the PD-OH and PD-NOH groups were observed in both early (H–Y stages 1–2) and advanced disease stages (H–Y stage >3), where PD-OH patients demonstrated substantially lower cognitive scores. In contrast, the intermediate stage (H–Y 2–3) showed only a non-significant trend toward lower cognition in PD-OH (SMD = −0.36, 95% *CI* [−0.73, 0.01], *p* > 0.05). Despite considerable heterogeneity within subgroups (*I*^2^ = 56–85%), formal testing confirmed no statistically significant differences between H–Y subgroups (*χ*^2^ = 2.37, *df* = 2, *p* = 0.31). This finding implies that, while disease severity modifies the magnitude of cognitive impairment in PD-OH patients, H–Y stage itself is not a primary determinant of the OH-cognition association. In the subgroup with a male proportion exceeding 50%, a significant difference between the PD-OH group and the PD-NOH group is manifested, with the mean value of the PD-OH group being significantly lower than that of the PD-NOH group; whereas in the subgroup with a male proportion less than 50%, no statistical significance was detected in the difference between the two groups. Nevertheless, in general, irrespective of the male proportion, a significant difference was present between the PD-OH group and the PD-NOH group in this indicator, and there was no significant difference in the magnitude of the difference between the two subgroups. In the subgroup analysis regarding education years, regardless of the length of education years, significant differences between the PD-OH group and the PD-NOH group in this indicator were consistently observed, with the mean value of the PD-OH group being significantly lower than that of the PD-NOH group. Although different levels of heterogeneity exist within each subgroup, no statistical significance was identified in the differences between subgroups, indicating that education years exert no significant influence on the results in this study. However, in terms of disease duration, significant differences between the PD-OH group and the PD-NOH group in the overall cognitive assessment scores are noted, and the difference between the two groups is more pronounced in the subgroup with a disease duration greater than 5 years. This finding implies that disease duration may be an important factor affecting the differences between PD-OH and PD-NOH, and the disparity between the PD-OH group and the PD-NOH group in this indicator becomes more evident as the disease duration prolongs.

**Table 3:** Subgroup analyses.

Subgroup analyses	*N*	SMD (95% *CI*)	*P*_heterogeneity_	*I*^2^ (%)	*P*_different_
Ethnicity					
Asian	8	−0.54 [−0.78, −0.31]	0.02	60%	0.82
Europe and America	5	−0.49 [−0.83, −0.16]	0.02	65%	
Disease duration					
>5 years	7	−0.65 [−0.89, −0.41]	0.03	57%	**0.04**
≤5 years	5	−0.30 [−0.52, −0.07]	0.2	34%	
H–Y stage					
1–2	4	−0.66 [−1.02, −0.29]	0.08	55%	0.31
2–3	5	−0.36 [−0.73, 0.01]	<0.01	71%	
>3	2	−0.79 [−1.25, −0.34]	0.19	43%	
Gender					
Men > 50%	11	−0.52 [−0.71, −0.32]	<0.01	63%	0.86
Men≤50%	2	−0.60 [−1.54, 0.33]	0.05	75%	
Education years					
>12 years	3	−0.78 [−1.26, −0.31]	0.09	58%	0.26
≤12 years	8	−0.46 [−0.76, −0.17]	<0.01	70%	

P_different_: Test for subgroup differences; P_heterogeneity_: *p* value for Cochran’s Q test. I^2^ (%): I^2^ statistic (I-squared statistic); N: Number of studies analyzed in the subgroup. P_different_ = 0.04; *p* < 0.05 for the test of subgroup differences indicates that disease duration (>5 years vs. ≤5 years) is a significant moderator of the observed effect, meaning the effect size differs significantly between these two subgroups.

#### 3.3.5 Bias analysis

**Figure 5:**
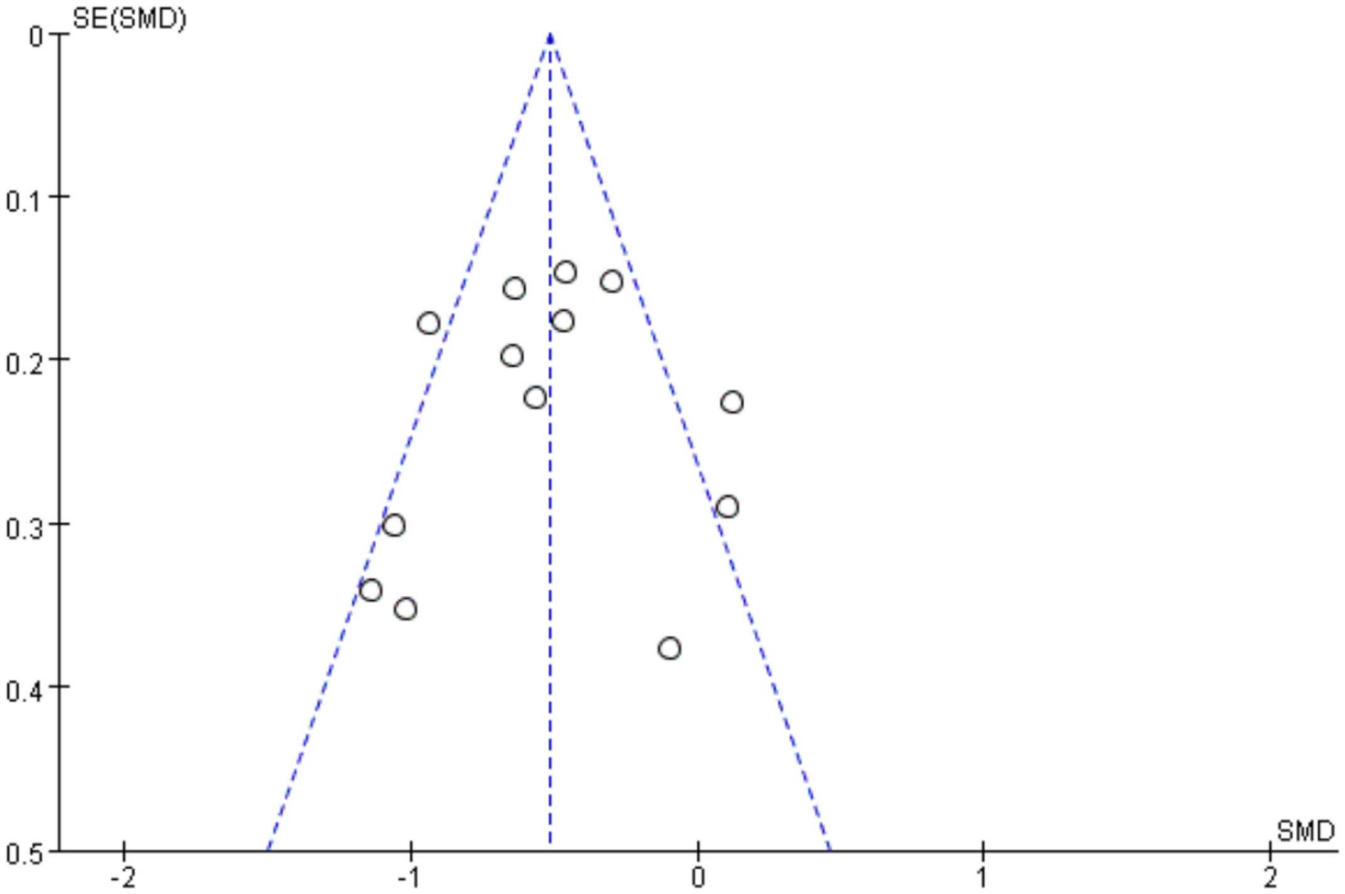
Funnel plots for the meta-analysis: cognitive differences in PD with vs. without orthostatic hypotension.

The funnel plot was used to investigate whether there was publication bias in this study, and the opposite side of the funnel plot meant that there was no publication bias. The funnel diagram of this study is shown in Figure 5. The result shows that the funnel diagram is symmetrical, and it is obtained by further Egger’s test, *p* = 0.79 ≥ 0.05 (Table 4). The studies were therefore included in the meta-analysis. Furthermore, no publication bias was detected, and the sensitivity analysis confirmed that the results were robust.

**Table 4:** Egger’s test.

Egger’s test					
Std_−_Eff	Coef.	Std. Err.	*t*	*p* > |t|	[95% *CI*]
Slope	−0.4082867	0.326305	−1.25	0.237	0.3099057
Bias	−0.5844212	1.584808	−0.37	0.719	2.903717

## 4 Discussion

This study delved into the relationship between OH and cognitive impairment in Parkinson’s disease through a systematic review and meta-analysis. The findings indicate a significant association between OH and cognitive decline in PD patients, particularly in the domains of executive, verbal, and visuospatial functions. This supports the notion that OH may serve as a marker for the progression of PD and the decline of cognitive function.

### 4.1 The relationship between global cognitive function and OH

The study revealed that, compared to PD patients without OH, the overall cognitive function scores of PD patients with OH were significantly lower (SMD = −0.62, 95%*CI* [−0.78, −0.46], *p* < 0.01). This result suggests that OH may be an important influencing factor in cognitive decline in PD patients, which is consistent with the conclusions of previous studies (43, 44). From a pathophysiological perspective, OH may accelerate cognitive decline through dual pathways: (1) direct hypoperfusion injury: Blood pressure fluctuations cause intermittent hypoperfusion in regions such as the entorhinal cortex and anterior cingulate cortex (ACC), disrupting neuronal functions related to reward processing, attentional control, and motivation regulation (45); (2) synergistic cerebral small vessel disease injury: *α*-synuclein-mediated autonomic dysfunction compounds OH-induced hypoperfusion, selectively damaging white matter watershed zones (46). This promotes white matter hyperintensity (WMH) accumulation and disrupts cortical–subcortical connectivity (4, 47). Notably, our subgroup analysis revealed a more pronounced cognitive impact of OH in PD patients with disease duration >5 years (SMD = −0.65 vs. SMD = −0.30 in the ≤5-year group, *p* = 0.04). This supports the ‘cumulative injury hypothesis’: chronic OH exposure likely synergizes with PD neurodegeneration through the aforementioned mechanisms, accelerating cognitive decline. These findings carry significant clinical implications: For long-duration PD patients, proactive OH management (e.g., individualized deprescribing of antihypertensives and increased fluid/salt intake) represents a viable strategy to mitigate cognitive deterioration. Future research should integrate ambulatory blood pressure monitoring with multimodal neuroimaging—such as arterial spin labeling (ASL) to quantify cerebral perfusion and diffusion tensor imaging (DTI) to assess white matter integrity—to further elucidate the spatiotemporal progression patterns of OH-mediated cognitive decline in Parkinson’s disease. Compounding this complexity, approximately 35% of PD-OH patients exhibit supine hypertension pathologically rooted in autonomic failure-induced blood pressure dysregulation (48). Critically, in PD patients with coexisting SH and OH, blood pressure variability contributes to cognitive dysfunction via cerebrovascular injuries (including white matter hyperintensities and cerebral microbleeds) and blood–brain barrier disruption, while nocturnal hypertension (manifested as a reverse dipping pattern) is associated with more severe cerebral damage (49). This “blood pressure turbulence” provokes greater microvascular spasm and accelerated white matter damage than isolated hypoperfusion, potentially explaining the significantly lower MoCA scores in PD patients with OH + SH vs. isolated OH (50–52).

### 4.2 Associations between cognitive domains and OH

In different cognitive domains, the PD-OH group showed significant cognitive impairment in executive, verbal, and visuospatial functions. Therefore, when PD patients are accompanied by OH, special attention should be paid to their cognitive impairment in executive function, verbal ability, and visuospatial function. Impaired executive function may be related to the dysfunction of the subfrontal cortical circuit (53). OH-induced cerebral hemodynamic changes may interfere with the normal functioning of this circuit. Verbal dysfunction may be related to insufficient blood supply to the brain’s speech centers, affecting speech expression and understanding. Visuospatial function deficits correlate with parieto-occipital hypoperfusion and angular gyrus microinfarcts, elevating spatial disorientation errors (54). Attention and memory domains were not significantly affected, perhaps because these cognitive functions involve more complex neural mechanisms and are relatively less affected by OH, or the currently included tests are not sensitive enough to early attention and memory impairment. These findings advocate for precision management: dual-phase BP control (midodrine for daytime OH + nocturnal antihypertensives for SH) in advanced PD (55); domain-targeted rehabilitation (e.g., visuospatial training for parieto-occipital impairment); and longitudinal biomarker studies integrating ambulatory BP monitoring with DTI/ASL-MRI to map the spatiotemporal progression of OH-mediated brain injury.

### 4.3 Comparison and analysis with previous studies

The results of this study showed a significant association between OH and global cognitive function decline, which was consistent with some previous studies (43, 44). However, in recent studies (56), the results were different in different cognitive domains. In the former study (56), OH was expected to correlate with the overall rating of the cognitive scale, but not with the observed results of the cognitive subdomain rating scale. In this study, five studies were included to compare different cognitive domains. Allcock et al. (30) divided 175 PD patients into the OH group and the non-OH group. After adjusting for the influence of age and equivalent dose of levodopa, the scores of attention and episodic memory in the OH group were lower than those in the non-OH group. Xue et al. (41) have shown that PD patients with OH have poor delayed recall memory, which may be due to the impaired dynamic cerebral autoregulation (dCA) ability leading to decreased metabolic function of the specific medial temporal lobe. Yin et al. (42) reported that, compared with the PD-NOH group, the PD-OH group had significantly higher scores in retelling ability. Centi et al. (31) have shown that, when the cognition of upright posture was assessed, executive function and memory deficits and visuospatial impairments were more common in PDOH. Pilleri et al. (37) have shown that, in neuropsychological assessments, OH patients showed impairments in specific cognitive tasks, such as the AttM test to explore sustained attention, the Corsi test to explore visuospatial working memory, and the RAVLT delayed recall to assess verbal memory. This difference may be due to the following aspects: (1) differences in research methods: Different studies have differences in case selection, diagnostic criteria, measurement tools, and sample size. For example, this study strictly limited the type of included studies to case–control studies and clearly stipulated the diagnostic criteria for OH and PD. Some previous studies may have used different study designs or had different diagnostic criteria, which may have led to inconsistent results; (2) differences in sample characteristics: The distribution of patients included in the study was different in terms of age, gender, course of disease, and ethnicity, which may affect the results. For example, the subgroup analysis in this study showed that the differences in cognitive function between the PD-OH and PD-NOH groups were more significant in patients with longer disease courses. Therefore, if the disease course distribution of the samples in previous studies is different, different conclusions may be drawn; and (3) control of confounding factors: Some potential confounding factors, such as comorbidity and drug therapy, may be controlled to different degrees in different studies. For example, certain drugs may affect both blood pressure and cognitive function, and if the use of these drugs is not properly controlled in studies, it may interfere with the true relationship between OH and cognitive impairment.

### 4.4 Advantages and limitations of the study

This study used a comprehensive literature search strategy, incorporating research from multiple Chinese and English databases, which maximally reduced the potential for publication bias. Strict inclusion and exclusion criteria, along with systematic literature quality evaluation, ensured the reliability and homogeneity of the included studies. Additionally, advanced meta-analysis methods were utilized to conduct a comprehensive data analysis. This meta-analysis has several limitations. First, to ensure comparability across studies, we only included research reporting outcomes assessed with the MMSE or MoCA. Studies utilizing other comprehensive cognitive tests were excluded from the meta-analysis, which may have limited the comprehensive assessment of cognitive domains affected by orthostatic OH. Second, the meta-analysis examining the association between MMSE or MoCA scores and OH showed a high degree of heterogeneity. Although sensitivity and subgroup analyses were performed to explore potential sources of this heterogeneity, the possible influence of unmeasured confounding factors and differences in cognitive assessment tools could not be fully excluded. Third, the included studies had relatively small sample sizes and geographical limitations. Although subgroup analyses suggested consistent cognitive deficits in PD-OH patients across ethnic groups, these results must be interpreted with caution due to critical imbalances in regional representation. Specifically, the ‘Europe and America’ subgroup predominantly reflected data from North American cohorts (four out of five studies), with only one European study included. This precludes meaningful conclusions about European populations and limits the generalizability of region-specific findings. Future multinational studies with balanced recruitment are warranted to validate potential geographical variations. Fourth, regarding the diagnosis of OH, it is noteworthy that the Active Standing Test is more commonly used in current studies than the Head-Up Tilt Test (57). Importantly, Chen-fei et al. (32) found that OH diagnosed by HUT is a stronger predictor of adverse clinical outcomes than OH diagnosed by AST. Therefore, the predominant reliance on AST in the included studies may have led to an underestimation of the true association between OH and cognitive impairment. Fifth, this study did not strictly distinguish between the effects on cognitive function caused by neurogenic OH vs. non-neurogenic OH, nor did it consider differences arising from acute vs. chronic OH. These mechanisms may have distinct clinical implications. Finally, despite using a comprehensive literature search strategy, the possibility of missing unpublished studies or negative results cannot be entirely ruled out, potentially introducing publication bias.

### 4.5 Clinical significance and prospect

The observed association between OH and cognitive impairment in PD patients underscores critical clinical implications. Clinicians should recognize the frequent co-occurrence of OH and cognitive deficits, particularly in patients with longer disease duration. Enhanced monitoring of executive, verbal, and visuospatial functions may facilitate the early detection of cognitive changes in PD-OH patients.

Future research could further explore the specific mechanisms by which OH leads to cognitive impairment in PD patients and develop more sensitive and specific biomarkers for early diagnosis and intervention. Meanwhile, more high-quality clinical studies should be carried out to evaluate the effectiveness and safety of comprehensive treatment strategies for OH and cognitive impairment, providing more robust evidence for the treatment of PD patients.

## 5 Conclusion

Cognitive decline in PD is associated with orthostatic hypotension. PD patients with OH demonstrate lower global cognitive scores than those without OH, exhibiting significant deficits in executive, verbal, and visuospatial functions—particularly among patients with longer disease duration. Clinicians should therefore actively screen for these potential cognitive impairments and implement comprehensive cognitive assessments alongside targeted interventions in PD patients experiencing OH.
